# Analysis of DNS Cache Effects on Query Distribution

**DOI:** 10.1155/2013/938418

**Published:** 2013-12-12

**Authors:** Zheng Wang

**Affiliations:** ^1^Computer Network Information Center, Chinese Academy of Sciences, No. 4 South 4th Street, Zhongguancun, Beijing 100190, China; ^2^China Organizational Name Administration Center, Jia 31, North Guangximen, Xibahe, Beijing 100028, China

## Abstract

This paper studies the DNS cache effects that occur on query distribution at the CN top-level domain (TLD) server. We first filter out the malformed DNS queries to purify the log data pollution according to six categories. A model for DNS resolution, more specifically DNS caching, is presented. We demonstrate the presence and magnitude of DNS cache effects and the cache sharing effects on the request distribution through analytic model and simulation. CN TLD log data results are provided and analyzed based on the cache model. The approximate TTL distribution for domain name is inferred quantificationally.

## 1. Introduction

The domain name system (DNS) is one of the most critical infrastructures of today's Internet [1], translating user-friendly host names into IP addresses.

The scalability of DNS relies heavily on the aggressive use of caching, which helps to cut client-perceived lookup delays and limit the wide-area network bandwidth consumption. For the top-level domain servers at the top of the DNS name space hierarchy, caching is especially advantageous for lowering the otherwise overwhelmingly high load. Compared with noncaching scenarios, DNS cache servers (mostly also called recursive servers) virtually play as proxies between the Internet users and the answering authoritative servers. Only the DNS requests from users missing the cache eventually arrive at the authoritative servers, while those hitting the cache should never be seen by the authoritative servers. Due to this kind of blinding effects, the original user query behaviors are hardly exposed to the authoritative servers and this tends to complicate or obscure the analysis of query behavior and the retrieval of query patterns.

It is commonly agreed that web traffic follows the Zipf-like distribution [2], which lays an analytical foundation for improving web access performance. Previous study shows that the noncached DNS request traffic also exhibits the Zipf-like distribution [3]. Given that the cache mechanism is widely employed in the DNS nowadays, we have no knowledge about how the DNS cache impacts the DNS query distribution. The analytical and measurement investigations of such effects are generally requisite for fully understanding the query load observed at authoritative servers and thereby facilitating the system improvements, performance enhancing, and protocol optimization efforts. The analysis presented in this paper is even more contributive to the area of DNS traffic modeling and monitoring, considering the facts that the query data collected at the user side are rarely available (mostly due to the privacy concerns), while those can be readily recorded at the authoritative servers. This work acts as an attempt to infer the user behavior through the modeling of the cache system utilizing authoritative server's query log.

There is hardly any previous work on the analytical explanation of DNS query distribution on authoritative servers, especially top-level domain servers. The most relevant work involves measurement studies on the DNS traffic at root servers [4], analysis of DNS resource records time-to-live (TTL) values at authoritative DNS server to balance DNS query rate at the desired level [5], and the effect of DNS caching perceived by Internet users [3]. But none of the work focuses on the bridging between traffic pattern of users and authoritative servers based on analysis of DNS caching. Our work seeks to fill the gap and make the DNS traffic more analyzable and understandable.

This paper is organized as follows. In Section 2, we filter out the five categories of malformed queries in the raw log file.  Section 3 presents the caching model and the theoretical results on the query distribution. In Section 4, based on the purified data, we give the popularity of domain names and infer their TTL distribution.

## 2. Filter Out Malformed DNS Queries

### 2.1. The Data

The DNS name space is hierarchical, where the upper level domain is administratively responsible for its lower level domains. Different domain levels play different roles, in both technique and policy. Top-level domain (TLD) is the highest level domain category in DNS. TLDs that are delegated for noncountry specific usage are called generic top-level domains (gTLDs). For example,  .com,  .net, and  .org are among the currently most popular gTLDs. Two-letter country code domains reserved for every country or region in the world is another type of TLDs, namely, country code top level domains (ccTLDs). Examples of ccTLDs include  .uk (United Kingdom), .us (United States), .ca (Canada), and  .cn (China). China Internet Network Information Center (CNNIC) takes the responsibility of China's domain name registry to operate and administrate “.CN” country code top-level domain (ccTLD) and Chinese domain Name (CDN) system.

There are currently 6 CN TLD name servers specified, with names in the form letter  .dns.cn, where letter ranges from a toe, pulsing ns.cernet.net. However, 4 of these 6 names have physical servers in multiple geographically distributed locations, using anycast address announcements to provide decentralized service. There are 15 anycast instances in 8 sites for CN TLD.

BIND is by far the most popular implementation of DNS today [1]. As a means for DNS diagnosis and analysis, BIND supports extensive logging, which consists of writing information to a debug file and sending information to syslog. All DNS query events, specified by the query category, are recorded in the log file by BIND. The log file is usually of the plain text format, which provides the raw DNS query data. A typical query record includes time, IP, port, domain name, and query type.

In this paper, we record query log by BIND at each CN TLD instance for approximate 24 hours from 22 March 2011, 22:54:44.474, to 23 March 2011, 23:06:39.727, and then accumulate them as a one-day's log. The total log file is over 200 GB and contains 1,047,586,432 queries.

### 2.2. Categorize Malformed Queries

Previous study observed a variety of problems with DNS queries [4, 6, 7], and here we refer to those kinds of DNS queries that should not occur on the wide-area Internet as malformed ones. As the malformed DNS queries actually pollute the Internet and have little, if not none, valuable information about the behavior of requesting domain names, we need firstly to filter out them to clarify the data set. We define the following five malformed query categories and describe what kind of characteristics the queries should have to match that category. Note that each query may be placed into more than one category. For example, the DNS log may contain a recursion desired query with an unknown TLD, thus qualifying for two categories. Our categories are as follows.

*Undelegated TLD.* This category refers to a top-level domain that is not officially delegated by ICANN. These invalid TLDs are found in the queries at CN TLD servers. Local misconfiguration is the main source of such TLDs, which is manifested by some local names like localhost, local, and corp. Other undelegated TLDs look like filename extensions, such as txt, html, and c.

*Recursion Desired*. There is a bit allocated in the DNS message, named recursion desired or RD [8]. It specifies whether the query requests recursive resolution service. While RD may be requested by any authoritative-only oblivious clients, the CN TLD servers as well as other TLD servers never provide recursive resolution service. For those RD requesters, the responses are always unsatisfactory since the responses are usually not the direct answers to the questions.

*Private Source IP*. Some IP addresses specified in RFC 1918 are reserved for private usages and should not be seen in the public network [9]. These private source IP addresses may be mistakenly leaked from the local area network traffic. Since the IP addresses are not globally routable, the responses are also unlikely to reach the requesters.

*Illegal Characters in Query Name.* The only valid characters in DNS names are the letters A–Z, numbers 0–9, and hyphen per the DNS protocol specifications [10]. While some DNS modifications have been standardized to accommodate the non-ASCII characters for languages other than English, such usages are quite limited till now. Moreover, such “internationalized domain names” should use the prefix “xn-.” In this paper, we filter out queries that contain characters outside the range defined by RFC 1035 [10].

*Non-CN TLD*. As mentioned above, CN TLD server is only responsible for CN domain name resolution. Therefore, resolvers should not request it for other TLDs. But actually we observed a nonignorable amount of non-CN DNS queries in the log. These non-CN TLDs include COM, NET, and ARPA. This would bring a waste of resources, as CN TLD server could do nothing but give a NXDOMAIN reply.

*Unused Query Class*. The DNS specifications only define five query classes: IN (1), CHAOS (3), HS (4), NONE (254), and ANY (255) [11]. As no other classes are defined, queries with unknown classes arriving at a CN TLD name server should be taken as malformed queries.

### 2.3. Filtered Data Results

According to the categories of malformed queries defined in Section 2.2, we filter out these categories with a self-written script. This leaves us with a few files containing all the queries of each category and another file containing remaining queries. We base our analysis on this legitimate data left as only this purified data is trustworthy for further study.

**Figure 1:**
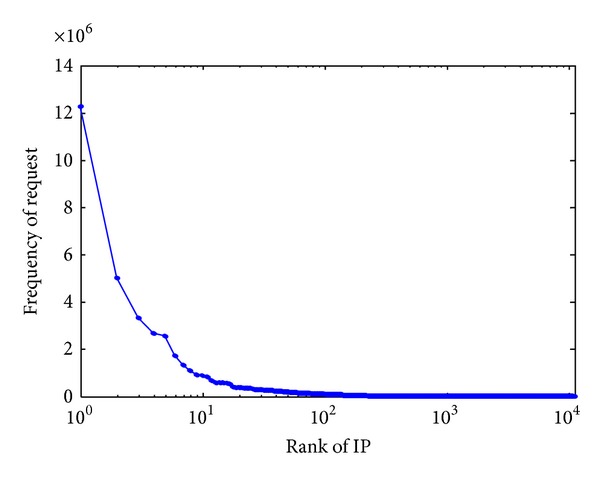
Frequency of request versus IP rank.

Table 1 shows the count and percentage breakdowns for the five query classifications defined in Section 2.2. Note that we found no unused query class in the data, which is different from previous study [4, 6, 7]. The legitimate data contain 896,525,985 queries from 11,153 unique source addresses and the distribution of number of queries per source is extremely skewed.  Figure 1 shows the source's rank (in the order of decreasing number of queries) on the *x*-axis and the number of queries per source on the *y*-axis. The number 1 ranking source alone sent 12,287,279 queries during the 24-hour observation period.

## 3. Models of DNS Resolutions

In this section, we present an overview of DNS, describe the resolution of a DNS system, and then model the DNS system as a whole for further analysis.

### 3.1. DNS Queries

The major task carried out by a name server is to respond to queries (questions) from a local or remote resolver or another name server acting on behalf of a resolver. A name server may have zone files that define it to be authoritative for some (if any) domains and slaves for others and may be configured to provide caching, forwarding, or other behaviors for other domains or users.

There are two types of queries defined for DNS systems.

*Recursive Queries*. The answer to a recursive query is the final answer to the question. This means that the receiving name server will complete all the relevant queries necessary for the final answer. This may involve contacting some authoritative name servers to obtain the intermediate referral information. Due to the security considerations, most authoritative name servers are not required to support recursive queries.

*Iterative Queries*. To answer an iterative query, the name server is not necessarily authoritative for the requested name. Only if the name server has any information useful for the final answer, it will return it. This information is called the referral information. Compared with recursive queries, the receiving name server is not required to make additional requests and return additional information if it has no useful information in itself. All name servers should at least support iterative queries.

**Figure 2:**
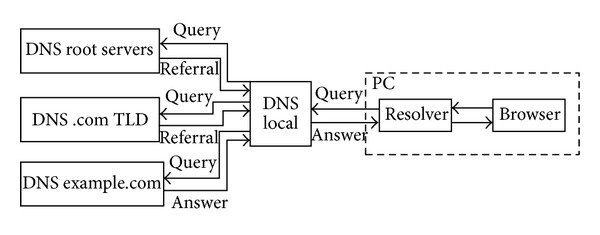
Recursive query resolution.

Figure 2 illustrates the typical recursive resolution mechanism. The client's browser is installed with a resolver, and the resolver requests a local recursive server for a name (e.g., example.com). If the query misses the DNS cache in this recursive server, the server will follow the steps in the figure to obtain the addresses for example.com. Requests will begin at well-known root servers in the DNS hierarchy. If the queried server has delegated its subzone containing the queried name, it returns a referral response. The response conveys the set of servers authoritative for the referral. The recursive server will choose one of these referral servers and repeat its question. This process typically proceeds until the final answer is returned by the authoritative server.

### 3.2. DNS Agent Assumptions

All DNS agents fall into one of two classes: stub and recursive. Stub resolvers are the simplest resolvers with the minimum functions. They rely on recursive resolvers that understand name server referrals. The implementations of recursive resolvers include the Berkeley Internet Domain Name (BIND) [1] server and Microsoft's DNS server. Except for a few public recursive DNS services, recursive resolvers are largely operated by organizations to serve their local clients. Recursive name servers can understand referrals and send iterative queries following these referrals. Recursive name servers may also cache the responses to save the lookup costs.

In this paper, we assume that only caching, recursive name servers talk to the authoritative servers or all queries come from caching servers. This assumption is logical for two reasons. Firstly, an overwhelming proportion of DNS queries do come from their local recursive name servers for these servers are the common Internet infrastructures in most areas. Secondly, most noncaching queries appear to come from stub resolvers and their RD bits are usually set by the stub resolvers. As discussed in Section 2, these RD queries are filtered out. Therefore, we can safely assume that all queries come from DNS agents which are caching name servers.

### 3.3. Models of DNS Resolutions

**Figure 3:**
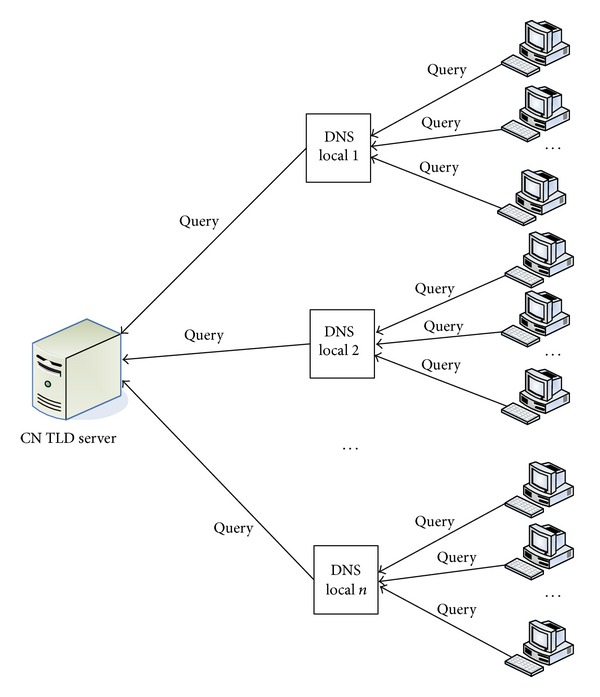
Model of DNS resolution.

According to the assumption of Section 3.3, all queries for CN domain name generated by client applications should arrive at the caching name server, and then these servers request the CN TLD server for the resolution answer on behalf of the initial query generator. Thus all queries, if not hit in the server cache, can reach the CN TLD server and be recorded by the DNS log.  Figure 3 shows that model.

To simplify the analysis of DNS query distribution, we assume that all clients share the common interests. So they produce queries for different CN domain names with the same proportion. At the same time, we allow that clients may have varied DNS traffic. The local caching name server talks to the CN TLD server for clients and thus acts as an agent as to the CN TLD server. Considering the different quantity of clients requesting local caching name servers, the clients' queries accumulated by the servers may also differ. What the CN TLD server sees is queries from a number of local DNS servers with hidden clients' requests.

## 4. Cache Effects of DNS Servers

In this section, we study an analytic model of DNS caching and formulate the hit and miss rate as well as the request rate for the CN TLD server. We also demonstrate the cache and cache sharing effects on query distribution.

### 4.1. Analytic Models

A simple analytic model for the cache query process is presented in [12]. Let the sequence of inter-arrival times of queries for the given data item be a sequence of independent and identically distributed (i.i.d.) random variables. This means that the interquery times are a renewal process.

**Figure 4:**
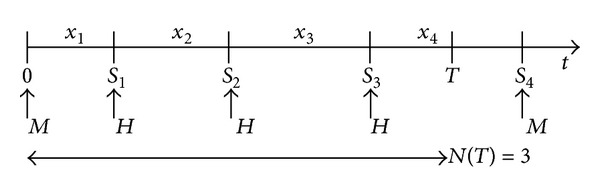
Renewal model for query process.

Figure 4 illustrates the idea. A cache miss happens at time *t* = 0. Subsequently, three queries come successively, at times *S*_1_, *S*_2_, and *S*_3_. These three queries are cache hits because they occur before the TTL expires at time *t* = *T*. The afterward fourth query at time *S*_4_ occurs after *t* = *T*, and therefore it is a cache miss. According to the renewal process analysis, let *N*(*t*) be equal to the number of queries for the given data item in the interval (0, *t*]. So, we have *N*(*T*) = 3, and the number of cache hits per miss is 3.

Let *H*(*T*) represent the limiting hit rate if the TTL is *T*. Then, the limiting miss rate, denoted by *M*(*T*), can be obtained. So, we have

(1) $$\begin{array}{c} H\left(T\right)=\frac{E\left[N\left(T\right)\right]}{E\left[N\left(T\right)\right]+1},\;\;M\left(T\right)=\frac{1}{E\left[N\left(T\right)\right]+1}. \end{array}$$

We assume poisson arrivals for the distribution of inter-request times. The request rate *λ* is defined to be the expected number of requests in a unit duration. So, we can have closed-form expressions for the hit rate and miss rate as a function of the TTL and *λ*. Assume there is one client requesting its local name server; then the hit and miss rate can be expressed as

(2) $$\begin{array}{c} H_{1}\left(T\right)=\frac{\lambda T}{\lambda T+1},\;\;M_{1}\left(T\right)=\frac{1}{\lambda T+1}. \end{array}$$

Usually, a local name server has more than one client (say, *n*). Let the clients' queries arrive at the local server independently and their request rates be the same as *λ*. Then according to the property of poisson process, the aggregated query arrival rates at the local server are *nλ*. Then, the hit rate and miss rate can be expressed as

(3) $$\begin{array}{c} H_{n}\left(T\right)=\frac{n\lambda T}{n\lambda T+1},\;\;M_{n}\left(T\right)=\frac{1}{n\lambda T+1}. \end{array}$$

### 4.2. Cache Effects on Query Distribution

As described in Section 3, the queries recorded in DNS log of the CN TLD server are actually the cache missed ones of local caching name servers. Such request rate for the CN TLD server *r*_TLD_ can be expressed as

(4) $$\begin{array}{c} r_{\text{TLD}}^{1}=\lambda *M\left(T\right). \end{array}$$

For multiple clients requesting a local server, (4) changes as follows:

(5) $$\begin{array}{c} r_{\text{TLD}}^{n}=n\lambda *M_{n}\left(T\right). \end{array}$$

One common characteristic in web workloads is the highly uneven distribution of references to files. In many cases, Zipf's law has been applied to model file popularity [2]. Zipf's law expresses a power-law relationship between the popularity *P* of an item (i.e., its frequency of reference) and its rank *r* (i.e., relative rank among the referenced items, based on frequency of reference). This relationship is of the form:

(6) $$\begin{array}{c} P\left(r\right)=\frac{c}{r^{\beta }}, \end{array}$$

where *c* is a constant and *β*, known as the *exponent* or *scaling parameter*, is often close to 1.

Previous studies show that the popularity of domain names also follows a Zipf-like distribution with *β* ≈ 0.91. Here, we analytically study what are the local DNS server's caching effects on the Zipf's-like distribution of popularity of domain names.

**Figure 5:**
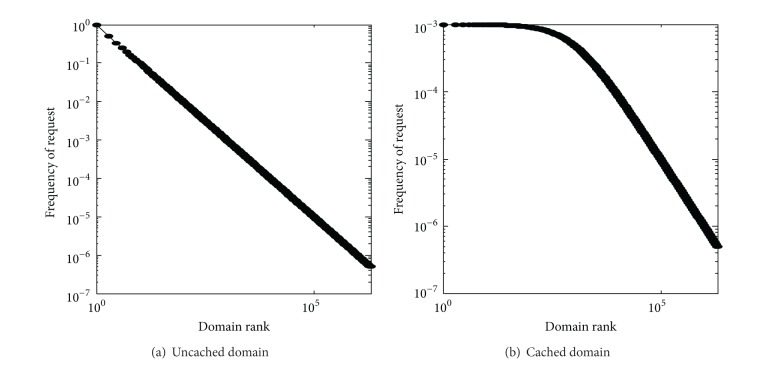
Caching effects on the domain name popularity.

The request rate for a domain name should be in direct proportion to its popularity; that is,

(7) $$\begin{array}{c} \lambda \left(r\right)=\frac{b}{r^{\beta }}, \end{array}$$

where *b* is a constant. We let *b*, the request rate for the most popular domain name, be 1,000 request/hour and the TTLs of all domain name resource records are all 1 hour. Let *β* = 1, 1 ≤ *r* ≤ 2,000,000.  Figure 5 shows the domain name popularity profile for the resulting cached queries for the CN TLD server. The primary impact of the DNS cache is to truncate or flatten the top-left portion of the original Zipf-like domain popularity distribution. In other words, most of the hits in the cache are for the highly popular domains that are rereferenced at short intervals.

### 4.3. Effect of Cache Sharing

The cache DNS name server has the aggregation effect on the original queries from its local clients. A request is not necessarily hit by the one cached from the same clients and it may be alternatively matched by the other clients' previous missed domain names. So, more local clients in a cache name server means higher cache hit rate and therefore more flattening effect on the domain name distribution.

**Figure 6:**
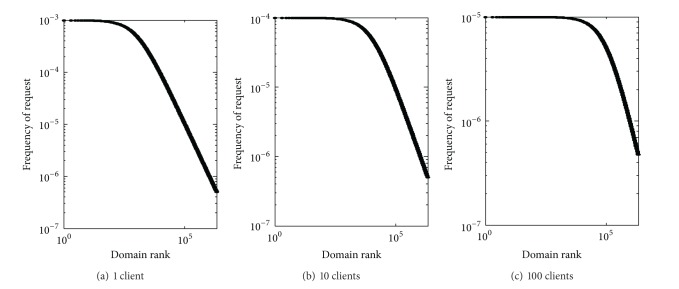
Caching sharing effects on the domain name popularity.

We vary the number of clients requesting a local name server from 1, 10 to 100 while holding the other parameters as shown in Section 4.2. The resulting domain name distributions are shown in Figure 6. As expected, only the leftmost portion of Figure 6(a) is almost flat, reflecting fairly uniform popularity of the most popular domain names seen by the CN TLD server. By contrast, the flattened portion is relatively larger in Figure 6(c) than in Figure 6(a), thanks to the cache sharing effect.

## 5. Log Data Results and Analysis

To deduce how the popularity of names varies in our CN TLD server log file, we plot query counts as a function of the popularity rank of a name. This graph, on a log-log scale, is shown in Figure 8.

In Figure 8, our observations are approximately consistent with a power law distribution. Granted that the query frequencies of domain names are independent and identically distributed, we give the maximum likelihood estimation (MLE) of the scaling parameter **β**. As a function of **β**, the likelihood function can be expressed as follows:

(8) L(β)=∑r=1Slog⁡p(r ∣ β)nr=−∑r=1Snr{βlog⁡r+log⁡∑r′=1Sr′−β}.

**Figure 7:**
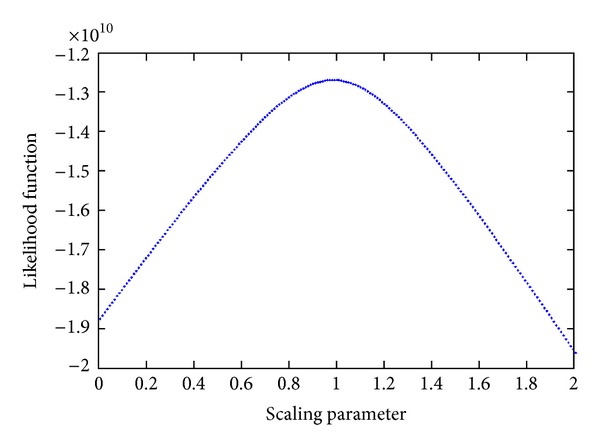
Likelihood function under different scaling parameters.

**Figure 8:**
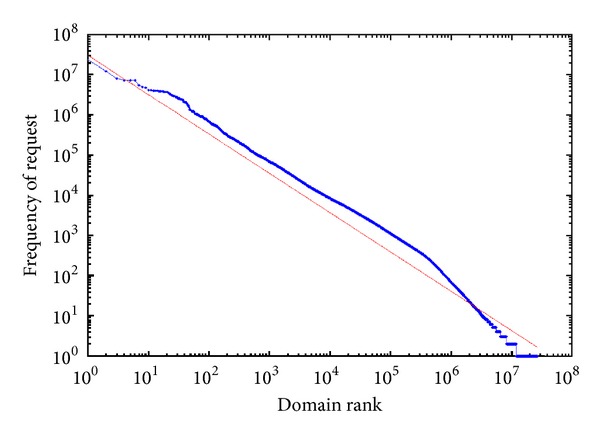
Popularity distribution of domain name.

Figure 7 is the likelihood function under different scaling parameter values. We can see that the maximum likelihood estimation is *β* ≈ 0.98, which indicates that the popularity of domain names follows a Zipf's distribution. The red line in Figure 8 is the fitting power law distribution with *β* = 0.98.

A typical DNS caching name server, usually the recursive one, has more than one hundred clients. Some large ISPs and organizations may even have thousands of clients requesting DNS cache service. So, the average conservative estimation of caching group size is 100. However, note that the distribution pattern differs markedly in Figures 6 and 8 (the initial straight line is better conserved in Figure 8 while severely flattened in Figure 6). Thus, the assumption of common TTL for Figure 6 seems unreasonable. Then, how TTL has effects on the distribution of domain popularity? Or what kind of TTL distribution just fits the curve in Figure 8? To answer this question we give the following theorem.

**Theorem 1**

Given that the request rate for a domain name follows a Zipf's-like distribution as (7) and the popularity of domain name observed at the CN TLD server also follows Zipf's-like distribution, the TTL distribution of domain name can be expressed as follows:

(9) $$\begin{array}{c} T=a*r^{\gamma }, \end{array}$$

where *a* is a constant and *γ* is very close to *β*.

**Proof**

According to (3), (4), and (7), we have the popularity of domain name observed at the CN TLD server as follows:

(10) $$\begin{array}{c} r_{\text{TLD}}^{}=\frac{b/r^{\beta }}{bT/r^{\beta }+1}. \end{array}$$

In order to make *r*_TLD_ display as Zipf's-like distribution, the denominator of the right side of (10) must be a constant for different popularity of domain names. That is,

(11) $$\begin{array}{c} \frac{T}{r^{\beta }}\approx a, \end{array}$$

where *a* is a constant. Therefore, the TTL distribution of domain name is (9) with *γ* very close to *β*.

The theorem tells how TTL values are distributed given how frequently each name is accessed. It is plausible that the more popular names have shorter TTL values, and then the corresponding effect on caching would be even more pronounced. Previous study shows that it is indeed the case that shorter-TTL names are more frequently accessed [3], which is consistent with the observation that DNS-based load balancing (the typical reason for low TTL values) makes sense only for popular sites. Based on the request rate for CN TLD server, we have inferred the approximate TTL distribution for domain name quantificationally.

Zipf-like distribution mainly comes from the request concentration. The load of CN TLD server is dominated by a small number of hot domain names. As this concentration is caused by the TTL's exponential-like distribution, we can enhance the cache effect by increasing the TLL value for the most popular domain names. However, this solution blocks DNS-based load balancing. Therefore, we suggest that DNS-based load balancing is not recommended for lowering the load of CN TLD DNS servers. Instead, there are alternative ways to perform load balancing by DNS. One possible way is to release the task of load balancing for the local DNS. The authoritative server answers the query with multiple name servers. Local DNS servers can use some algorithm to spread the load and therefore ensure the desired result. For instance, it can provide each name server once in a round-robin sequence weighted by some factors.

## 6. Conclusions

Our study shows that DNS query for the CN TLD server follows Zipf-like distribution. Through the analytic model and analysis of cache effects, we have inferred the TTL's exponential-like distribution. This distribution lays out a theoretical foundation to improve current DNS systems for lowering the load of DNS CN TLD servers.

## Figures and Tables

**Table 1:** CN Malformed query classification.

Type	Count	Percent (/legitimate)	Percent (/total)
Legitimate	896,525,985	100	85.60
Undelegated TLD	39,552	0.005	0.004
Non-CN TLD	125,547,431	13.99	11.98
Recursion desired	2,262,502	2.52	2.16
Private source IP	15,713,796	1.23	1.05
Illegal name	1,208,211	0.35	0.30

Total	1,047,586,432	116.82	100
